# ULTRASOUND-GUIDED GENICULAR NERVE BLOCK FOR KNEE OSTEOARTHRITIS: A CASE SERIES

**DOI:** 10.1590/1413-785220243203e277781

**Published:** 2024-07-26

**Authors:** Ramon Chaves Ramalho, Tifani Dawidowicz Fernandes, Felipe de Freitas Peraro, Gabriel Marques Pugliese, Gustavo Gonçalves Arliani, Gabriel Ferraz Ferreira

**Affiliations:** 1. Instituto Prevent Senior, São Paulo, SP, Brazil

**Keywords:** Nerve Block, Pain, Knee Osteoarthritis, Ultrasonography, Anesthetics, Bloqueio Nervoso, Dor, Osteoartrite do Joelho, Ultrassonografia, Anestésicos

## Abstract

**Objective:**

Knee genicular nerve blocks have been a topic of discussion among various types of treatment for knee osteoarthritis. This study aims to evaluate the pain and function of patients diagnosed with knee osteoarthritis after undergoing ultrasound-guided genicular nerve blockade using pharmacological agents.

**Methods:**

The study included 36 patients diagnosed with knee osteoarthritis, comprising 17 bilateral cases, totaling 53 knees undergoing UGNB using a mixture of triamcinolone, ropivacaine, and lidocaine under ultrasound guidance. Epidemiological data, pain outcomes measured by the Visual Analog Scale (VAS), and function assessed using the Western Ontario and McMaster Universities (WOMAC) score were evaluated before and after 12 weeks of the procedure.

**Results:**

The mean age was 75.5 years (standard deviation of 9.4 years), with a predominance of females and right-sided involvement. There was a mean reduction of 3.0 points in VAS (p < 0.001) and 15.4 points in WOMAC (p < 0.001). Two cases reported only minor and transient complications related to the procedure (skin anesthesia and edema).

**Conclusion:**

Ultrasound-guided genicular nerve blockade using pharmacological agents demonstrated pain reduction and improved function with a low complication rate after 12 weeks in patients with knee gonarthrosis. *Level of Evidence IV, Case Series.*

## RESUMO

**Objetivo:**

Os bloqueios geniculares do joelho têm sido tema de discussão entre os diversos tipos de tratamento da gonartrose. Este estudo tem por objetivo avaliar a dor e a função dos pacientes com diagnóstico de osteoartrose do joelho, após realização do procedimento de bloqueio farmacológico dos nervos geniculares (BFNG) guiado por ultrassom.

**Métodos:**

O estudo incluiu 36 pacientes com diagnóstico de gonartrose, sendo 17 casos bilaterais, totalizando 53 joelhos submetidos ao BFNG, com a mistura de triancinolona, ropivacaína e lidocaína guiado por ultrassom. Avaliou-se dados epidemiológicos, desfechos de dor pela Escala Visual Analógica (EVA) e função com escore Western Ontario and Mcmaster Universities (WOMAC) antes e após 12 semanas do procedimento.

**Resultados:**

A idade média encontrada foi de 75,5 anos (desvio padrão de 9,4 anos), com predominância do sexo feminino e do lado direito. Houve uma redução média na EVA de 3,0 pontos (p < 0,001) e no WOMAC de 15,4 (p < 0,001). Em dois casos, relataram apenas complicações menores e transitórias relacionadas ao procedimento (anestesia da pele e edema).

**Conclusão:**

O bloqueio farmacológico dos nervos geniculares guiado por ultrassom demonstrou redução da dor e melhora na função, com baixa taxa de complicação após 12 semanas nos pacientes com gonartrose. Nível de evidência IV, Série de Casos.

## INTRODUCTION

 Knee osteoarthritis or gonarthrosis is the main cause of knee pain, affecting about 10% of the world population aged over 60 years, mainly women. ^1^ It is characterized by continuous mechanical stress associated with the local inflammatory process, causing wear in the intra-articular structures of the knee. As a result, the knee can be swollen, with limited range of motion, and in more advanced cases even evolve with deformities. ^2^

 Total knee arthroplasty can be a successful surgical option for cases that do not respond to conservative treatments, but with risks and complications already well described in the literature and, thus, patients often request less invasive interventions before accepting arthroplasty. ^3^

 The diagnosis is clinical and is complemented by imaging exams, with weight-bearing knee radiography being the main exam. In this examination, there is usually a decrease in joint space, osteophytes and subchondral sclerosis. ^4^ To monitor the progression of the disease and aid in treatment, the Ahlbäck radiographic classification can be used. ^5^

 The initial treatment is conservative through weight loss, physical therapy and the use of pain relievers. In advanced cases, where patients have functional limitation and no improvement with lifestyle changes, more invasive interventions such as infiltrations, blocks and surgery are indicated. Even after all these procedures, patients may still develop refractory pain. ^6^

 Current studies demonstrate that genicular nerve blocks (GNB) using pharmacological agents, such as corticosteroid and anesthetic solutions, can relieve pain and improve the patient’s functional capacity. ^7^ In patients with gonarthrosis, the intervention is performed on the sensory branches of genicular nerves: superomedial, superolateral and inferomedial. ^8^

The objective of the study was to compare the clinical pain and function outcomes of patients with knee osteoarthritis submitted to ultrasound-guided genicular nerve block using pharmacological agents.

## MATERIAL AND METHODS

The present study is a retrospective case series of patients diagnosed with knee osteoarthritis submitted to ultrasound-guided GNB.

The project was approved by the Local Ethics Committee (Brazil Platform - CAAE: 72636023.0.0000.8114). The research followed the principles of the Declaration of Helsinki and was based on the guide on good clinical practices at all stages.

### Inclusion and exclusion criteria

Inclusion criteria: patients with diagnosis of gonarthrosis, clinical and radiographic, refractory to conservative treatment and who were submitted to pharmacological ultrasound-guided genicular nerve block by the senior author (G.F.F.) between June 2022 and February 2023.

Exclusion criteria: patients with previous knee surgeries or fractures; individuals with autoimmune diseases such as rheumatoid arthritis, lupus, among others.

### Evaluated outcomes and follow-up

 Patients were clinically evaluated through the pain score using the Visual Analogue Scale (VAS) ^9^ and the *Western Ontario and McMaster Universities* (WOMAC) function score. ^10^ Patients were evaluated at two times: before the procedure and 12 weeks after the block. 

 The Ahlbäck classification was used to measure the degree of knee osteoarthritis before the intervention. This classification was initially published by Ahlbäck in 1968 ^11^ and later revised in 1992 by Keyes et al. ^12^

This stratification is based on the weight-bearing radiographic view of the knee.. The score ranges from grade 1 to grade 5. Grade I: decreased joint space; Grade II: obliteration of joint space; Grade III: anteroposterior view indicates tibial plateau wear of less than 5.0 mm and profile view shows intact posterior part of tibial plateau; Grade IV: anteroposterior view shows tibial plateau wear between 5.0 and 10.0 mm, and profile view shows extensive wear of posterior margin of plateau; Grade V: anteroposterior view shows severe tibial subluxation, and profile view shows anterior tibial subluxation greater than 10.0 mm.

### Data collection

Data were collected from patient medical records and also included information such as age, sex, laterality, height, weight, presence of occurrences or complications resulting from the procedure, and calculated Body Mass Index (BMI).

 Study data were entered and managed using Research Electronic Data Capture (REDCap) tools hosted at the Prevent Senior Institute. REDCap is a secure, web-based application designed to support data capture for research studies by providing: 1) an intuitive interface for validated data entry; 2) audit trails for data manipulation tracking and export procedures; 3) automated export procedures for continuous data downloads for common statistical packages; and 4) procedures for importing data from external sources. ^13^

### Procedure and intervention

The entire procedure was performed exclusively in an outpatient setting. For GNB, patients were initially positioned in horizontal dorsal decubitus on the stretcher with a pad in the popliteal region to leave in slight flexion and the knee asepsis and antisepsis were performed.

The pharmacological solution used was composed of 2.5 mL ropivacan (7.5 mg/ml), 2.5 mL lidocan (2%) without vasoconstricting component and 1.0 mL triamcinolone. The solution totaled 6.0 mL and 2.0 mL were applied to each genicular nerve: superolateral, superomedial and inferomedial.

 The entire procedure was guided by Toshiba Aplio300 ^®^ Ultrasound with linear transducer, protected by sterile cover. Initially, we located the joint on the long axis, and then we looked for the genicular bundle, best visualized by pulsing each genicular artery. Using a 22G spinal anesthesia needle, the drug was delivered to the correct region by needling in plane and direct visualization. 

The procedure was always performed by the same physician, experienced in guided intervention (G.F.F.), following the standard of medication and application for all patients in the study. Inferolateral genicular nerve block was not performed in order to avoid iatrogenic injury or block of the common peroneal nerve

After the procedure, the patient was immediately referred for a consultation and evaluation by the physiotherapy team to guide the entire rehabilitation process specific to each patient.

### Statistical analysis

Statistical data analysis was performed through the continuous variables that passed the Shapiro-Wilk normality test. For comparisons of data distributed in a non-parametric way, the Wilcoxon test was used. For normally distributed data, the paired Student’s t-test was used. Categorical variables were evaluated by their proportion. Subgroup analysis was performed through a linear regression comparing the pain scale (VAS) and the degrees of arthrosis (Ahlbäck). All statistical evaluations were performed using the R software. Statistical evidence was considered when p-value ≤ 0.05.

## RESULTS

The present study analyzed 36 patients, totaling 53 knees (17 bilateral), most on the right side (52.8%) and with a predominance of females with 83% of the sample. The mean age of the patients analyzed was 75.5 years, with a minimum age of 57 years and a maximum age of 95 years, with a standard deviation (SD) of 9.4 years. The mean BMI of the included patients was 29.7 (SD 4.2).

Regarding the degree of knee osteoarthritis, most patients (38%) were classified as grade II, 34% as grade III, 18% as grade IV and 10% as grade V.

**Figure 1.**
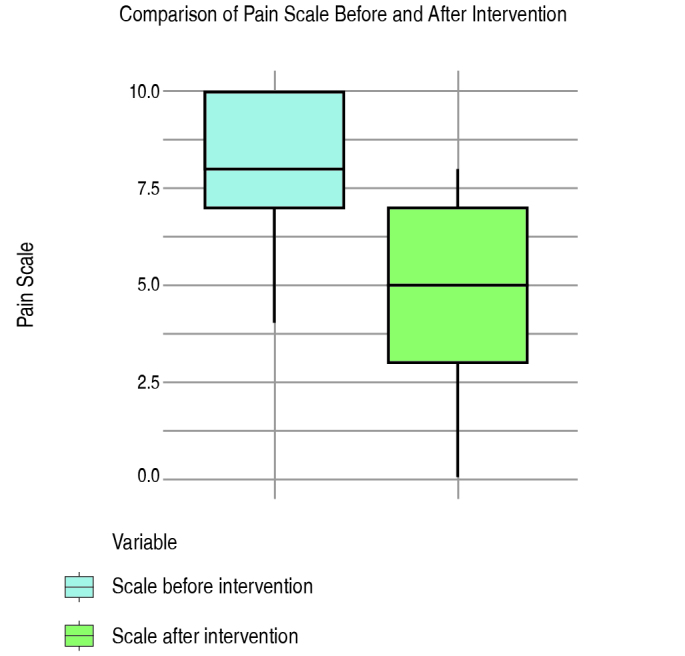
Comparison of VAS before and after intervention

 Regarding the clinical evaluation, there was a mean reduction of 3 points in the visual analogue scale of pain after pharmacological block. The mean initial value was 8.0 points (SD 1.6) and after the block with 12 weeks of follow-up the VAS decreased to 5.0 (SD 1.2), obtaining a 3.0-point reduction in the mean in relation to the initial value (p < 0.001), as shown in Figure 1 . 

 Regarding the WOMAC score, there was a 15.4-point reduction (p < 0.001). In this same score, sub-items were analyzed with a reduction of 4.1 points for pain (p < 0.001), a reduction in joint stiffness of 1.1 point (p < 0.05) and in function of 7.2 (p < 0.001), showing a positive impact on the different dimensions evaluated by the WOMAC score ( Table 1 ). 

**Table 1.** Pre- and post-intervention clinical evaluation results.

Outcome	Pre-intervention (mean, standard deviation, minimum and maximum)	Post-intervention (mean, standard deviation, minimum and maximum)	Pre- and post-intervention mean difference	p-value
VAS ^*^	8,0 ± 1,6 [4,0-10,0]	5,0 ± 2,1 [0-8,0]	-3,0	p < 0,001
WOMAC ^**^	64,1 ± 16,7 [25,6-93,7]	48,7 ± 25,1 [2,0-93,8]	-15,4	p < 0,001
WOMAC (Pain)	12,7 ± 3,2 [6,0-18,0]	8,6 ± 5,1 [9-20,0]	-4,1	p < 0,001
WOMAC (Joint Stiffness)	4,1 ± 2,1 [0-8,0]	3,0 ± 1,4 [0-6,0]	-1,1	p < 0,05
WOMAC (Function)	45,3 ± 12,3 [15,0-67,0]	35,4 ± 18,6 [0-68,0]	-7,2	p < 0,001

* Visual Analogue Scale;

** Western Ontario and McMaster Universities

**Figure 2.**
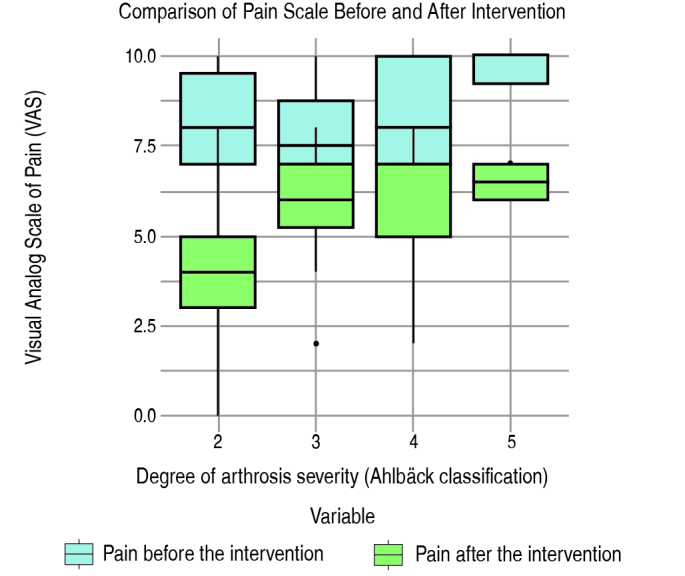
Comparison of VAS before and after intervention considering the degree of Ahlbäck

### Subgroup analysis

 The subgroup analysis of VAS before and after intervention stratified by the degree of osteoarthritis (Ahlbäck classification) showed that the coefficient for the degree of osteoarthritis is statistically significant (p = 0.00467). This suggests a significant relation between degree of osteoarthritis (Ahlbäck) and VAS 12 weeks after genicular nerve block, suggesting that the severity of osteoarthritis may influence the response to therapy ( Figure 2 ). In this case, the lower the severity of arthrosis, the greater the reduction in pain 12 weeks after genicular nerve block. 

### Complications

Of the 53 cases where block was performed, only two (3.8%) presented complications, and the two complications were considered minor. One patient had edema in the knee region and another had loss of sensation on the lateral face of the knee, both temporary. There were no major complications.

## DISCUSSION

 It is known that the initial treatment of knee osteoarthritis is conservative, with physiotherapy, non-steroidal anti-inflammatory drugs (NSAIDS) and analgesics. ^6^

 NSAIDS and analgesics are indicated for patients with mild to moderate osteoarthritis, while physiotherapy is indicated for strengthening the quadriceps, flexibility and improvement of physical fitness, serving as an aid for drug treatment. ^14^ ^,^ ^15^

 As the condition becomes more complex, other therapeutic methods are employed, such as injectable corticosteroids, intra-articular hyaluronic acid, and, more recently, genicular nerve block. ^16^ ^-^ ^19^

 Injections are reserved for patients with low response to oral medication, and joint infiltrations with hyaluronic acid have seen increasing adoption over the years. ^20^ However, the outcome in more advanced cases of knee osteoarthritis is often insufficient to relieve symptoms. 

Thus, GNB is a therapeutic option for knee osteoarthritis in order to relieve pain and allow a window of opportunity for rehabilitation, often compromised by pain.

Patients in our sample reported an important reduction in pain symptoms 12 weeks after the procedure, with a 3-point decrease in the mean value of the visual analogue scale of pain (p < 0.001) and in the WOMAC score of pain (p < 0.001).

There was also a relation between the power to reduce pain and the degree of osteoarthritis, that is, the greater the joint destruction, the lower the power of GNB. This reinforces the fact that advanced degrees of knee osteoarthritis have a worse result when compared to milder degrees.

In addition, we observed an improvement in relation to the stiffness measured in the WOMAC score, with lower significance when compared to other sub-items (p < 0.05). This is probably due more to the perception of improved pain than to an increased range of motion, as GNB does not reach the joint region.

In these 12 weeks, with improved pain and function, patients have the ideal time to perform intense and individualized physiotherapy rehabilitation, avoiding the recurrence of symptoms and the need for a new block. However, it is important, before the intervention, to explain this concept to the patient and the need for rehabilitation programs that should be followed subsequently.

 Some studies on GNB have been published. In the article of Kim et al. ^2^ the combination of GNB with lidocaine and corticosteroids provided short-term pain relief, although the contribution of corticosteroids was not clear compared to local anesthesia alone (control group). In the present study, the findings are similar, emphasizing the effectiveness in relieving pain in patients with knee osteoarthritis. 

 In the study conducted by Shanahan et al. ^16^ the authors published a 12-week placebo-controlled clinical trial to investigate the effects of GNB in patients with knee osteoarthritis. In the study group, patients received corticosteroid and bupivacaine block. Comparing those results with our findings, there was also an improvement in pain and function after genicular nerve block. 

 Tan et al. ^21^ conducted a systematic review on ultrasound-guided GNB for chronic knee osteoarthritis. They analyzed nine studies that included a total of 280 patients with symptoms or characteristics of the disease for at least 3 months. 

The studies used different block techniques and pharmacological agents, such as local anesthetics, corticosteroids and alcohol. The review showed sustained improvements in knee pain and function for up to 6 months after the procedure, regardless of the choice of pharmacologic agents.

Although it was not possible to perform a meta-analysis due to the heterogeneity of the studies, techniques and agents, it was concluded that there is solid evidence to target the upper medial and lower medial genicular nerves with local anesthetics, corticosteroids or alcohol, resulting in reduced pain and improved function in patients with chronic knee osteoarthritis under ultrasound guidance.

### Generalization

This study was conducted with a population with a mean age of 75.5 years and the generalization of the results for younger ages should be performed with caution.

### Study limitations

First, it is a case series without a control group. Second, the study was retrospective and with data collected from patient medical records. Finally, the mean age of the study was above the population with osteoarthritis, limiting the result in younger populations.

### Summary of evidence

The present study demonstrated that ultrasound-guided genicular nerve block in patients with knee osteoarthritis showed improved pain and function in the short term with a low rate of complications.

## CONCLUSION

Ultrasound-guided genicular nerve block demonstrated a reduction in pain and improvement in function with low rate of complications after 12 weeks in patients with knee osteoarthritis.
